# Slope Entropy: A New Time Series Complexity Estimator Based on Both Symbolic Patterns and Amplitude Information

**DOI:** 10.3390/e21121167

**Published:** 2019-11-28

**Authors:** David Cuesta-Frau

**Affiliations:** Technological Institute of Informatics, Universitat Politècnica de València, Alcoi Campus, 03801 Alcoi, Spain; dcuesta@disca.upv.es; Tel.: +34-966528505

**Keywords:** permutation entropy, sample entropy, signal classification, symbolic dynamics, discriminating power

## Abstract

The development of new measures and algorithms to quantify the entropy or related concepts of a data series is a continuous effort that has brought many innovations in this regard in recent years. The ultimate goal is usually to find new methods with a higher discriminating power, more efficient, more robust to noise and artifacts, less dependent on parameters or configurations, or any other possibly desirable feature. Among all these methods, Permutation Entropy (PE) is a complexity estimator for a time series that stands out due to its many strengths, with very few weaknesses. One of these weaknesses is the PE’s disregarding of time series amplitude information. Some PE algorithm modifications have been proposed in order to introduce such information into the calculations. We propose in this paper a new method, Slope Entropy (SlopEn), that also addresses this flaw but in a different way, keeping the symbolic representation of subsequences using a novel encoding method based on the slope generated by two consecutive data samples. By means of a thorough and extensive set of comparative experiments with PE and Sample Entropy (SampEn), we demonstrate that SlopEn is a very promising method with clearly a better time series classification performance than those previous methods.

## 1. Introduction

The capability of entropy or complexity measures to distinguish among time series classes and to understand the underlying dynamics is very well known [1,2,3]. Many different formulas, statistics, algorithms, or methods have been proposed since the introduction of the first method that arguably became widespread and generally used across a varied and diverse set of scientific and technological frameworks, Approximate Entropy, ApEn [4].

Most of these methods are based on counting events found or derived from the input time series under entropy analysis, in order to estimate probabilities from relative frequencies of such events. These probabilities are finally mapped to a single value that supposedly accounts somehow for the dynamic behaviour of the time series. These mapping frequently takes place using entropy definitions, such as Shannon [5], Renyi [6], Tsallis [7], or Kolmogorov–Sinai [8] entropies, among others not so often used.

The development of new entropy quantification methods is an ongoing and fruitful process. Since the introduction of ApEn, other derived methods have been proposed, such as Sample Entropy (SampEn) [9], Fuzzy Entropy (FuzzyEn) [10], Quadratic Sample Entropy (QSE) [11], and many more based on counting pattern matches in terms of time series subsequences amplitude differences. In addition, characterization studies related to these measures have also been published in order to avoid blind application of such methods or to maximise the performance achieved [12,13,14,15,16]. The concomitant analysis of time series at different temporal scales is also a frequent strategy in practically any method to gain a better insight into temporal dynamics [17,18].

Another successful line of research is also based on counting pattern matches but using a symbolic representation of a time series subsequence instead of its original sample amplitude form. A good representative of this approach is the Lempel–Ziv Complexity (LZC) [19], but due to its simplicity and robustness, Permutation Entropy (PE) [20] is probably becoming the most used entropy measure in this group, well above LZC. There are also additional studies devoted to the characterization of these symbolic methods [21,22,23,24,25,26] and to implement multiscale temporal analyses [27,28].

As stated above, PE is a very successful entropy statistic, but despite its strengths, it still has a few, however important, weaknesses. Since PE is based on relative frequencies of ordinal patterns resulting from sorting subsequences, when equal values are found in such subsequences, there is an ambiguity in the sorting process that has to be addressed consistently. These equal values or ties can lead to a misinterpretation of the time series nature [26], although they seem to play a minor role where classification tasks are concerned [24]. Regardless, several methods have been proposed to address this potentially detrimental weakness [29,30].

It has also been frequently claimed that not including amplitude information could have an adverse impact on PE performance, too. Thus, PE variations that consider both ordinal and amplitude information have been proposed, such as Weighted-PE [31], Amplitude–Aware-PE [29], or Fine Grained-PE [32]. In this case, the inclusion of amplitude information does seem to improve the discriminating power of PE in classification tasks [33].

Due to this significance, we tried to devise another method that also combined a symbolic representation of patterns and amplitude information. We based our idea on methods employed for syntactic pattern recognition and polygonal approximation of data, successfully used in the past to classify electrocardiogram (ECG) records [34,35]. The basic idea was to encode the magnitude of the amplitude differences between consecutive samples in the time series by an alphabet in which symbols accounted for a range of differences, spanning from 0 to ∞. The method should also be simple, efficient in terms of memory requirements and computational cost, and without a strong dependence on thresholds and parameters.

Based on the scaffolding provided by the standard PE algorithm, we propose in this paper a new entropy statistic termed Slope Entropy (SlopEn) that satisfies the requirements stated above. The method uses an alphabet of three symbols, 0, 1, and 2, with positive (+) and negative versions (−) of the last two. Each symbol covers a range of slopes for the segment joining two consecutive samples of the input data, and the relative frequency of each pattern found is mapped into a real value using a Shannon entropy approach [5].

In order to validate the approach proposed, a comprehensive experimental comparative study was conducted. The comparison took place using PE (ordinal patterns) and SampEn (amplitude patterns), two of the most representative entropy measures used in the scientific literature, and an experimental dataset based on publicly available records to ensure reproducibility. The results confirmed SlopEn as an entropy measure of great potential that outperformed both PE and SampEn under a great disparity of conditions and experimental settings.

## 2. Materials and Methods

This study uses two well known entropy methods, PE and SampEn, as the references against which the new SlopEn method can be validated. The input time series is referred to as the vector $x=\left\{x_{0},x_{1},\ldots ,x_{N-1}\right\}$, where $x_{i}$ is the i−th amplitude sample, and the number of samples is *N*. The embedded dimension for all measures is referred to as *m*.

The experimental dataset contains synthetic and real records. This dataset has been chosen with two purposes in mind: facilitate the reproducibility of the results by using publicly available data and analyse records difficult to classify by the standard PE method. It also contains not only biomedical data but electricity consumption data, as well as other records successfully classified by PE, in order to offer a complete and unbiased picture of the SlopEn capabilities and improvements. The methods stated above and the dataset are described in the next sections.

### 2.1. Sample Entropy

SampEn [36] is based on computing the relative frequency of similar amplitude subsequences. A subsequence starting at sample *j*, of length *m*, defined as $x_{j}^{m}=\left\{x_{j},x_{j+1},\ldots ,x_{j+m-1}\right\}$, is compared with all the other possible subsequences of length *m* starting at sample *i*, extracted from x, except with itself, that is, 0≤i,j<N−m+1,i≠j.

The distance between $x_{j}^{m}$ and $x_{i}^{m}$ is given by $d_{ji}=\max(|x_{j+k}-x_{i+k}\left|\right),0\leq k\leq m-1$. In order to consider two subsequences similar, this distance should be below a predefined threshold, usually termed *r*. In this work, *r* was set to 0.25 in all the experiments.

The number of subsequences similar to $x_{j}^{m}$ is stored in a specific counter, $B_{j}^{m}\left(r\right)$. When all the possible subsequences in x have been processed, a final statistic for the time series is computed as:

$$B^{m}\left(r\right)=\frac{1}{N-m}\sum_{j=0}^{N-m-1}B_{j}^{m}\left(r\right)$$.

The length of the subsequences is then increased by 1, and the previous similarity calculations are repeated for this new length. In this case, the final statistic is termed $B^{m+1}\left(r\right)$. SampEn can then be computed as:

(1) $$\mathrm{SampEn}\left(x,m,r,N\right)=-\log\left[\frac{B^{m+1}\left(r\right)}{B^{m}\left(r\right)}\right].$$

### 2.2. Permutation Entropy

PE [20] is based on computing the relative frequency of ordinal patterns associated to time series subsequences. As for SampEn, all possible subsequences $x_{j}^{m}$ are sequentially drawn from x. Then, the samples in $x_{j}^{m}$ are sorted in ascending order. The original indices of these samples conform another vector featuring the final location of each sample once they were sorted. This vector is usually defined as $\pi_{j}^{m}=\left\{\pi_{0},\pi_{1},\ldots ,\pi_{m-1}\right\}$ such that $x_{j+\pi_{0}}\leq x_{j+\pi_{1}}\leq x_{j+\pi_{2}}\ldots \leq x_{j+\pi_{m-1}}$. The number of different ordinal patterns that can emerge from an alphabet of *m* symbols, is m!. Thus, comparing the ordinal pattern found, $\pi_{j}^{m}$, with a list of all the possible m! ordinal patterns, it is possible to compute the relative frequency of each one. Thus, if a certain ordinal pattern has been found $c_{k}$ times, its relative frequency can be obtained as $p_{k}=\frac{c_{k}}{N-m+1}$. PE can then be computed as the Shannon entropy of the estimated probabilities:

(2) $$PE=-\sum_{k=0}^{m!-1}p_{k}\log\;p_{k},\forall p_{k}>0.$$

### 2.3. Slope Entropy

The purpose of SlopEn is to somehow include amplitude information in an otherwise symbolic representation of the input time series. Similar approaches have used a linear quantization scheme, with as many thresholds as levels desired, being Lempel–Ziv Complexity (LZC) [19] a good and generic representative of this approach, based usually on a single threshold and two symbols, 1 and 0. However, these methods are usually very dependent on the specific threshold chosen, and on the amplitude range of the time series under analysis.

Symbolic dynamics is a field of research that has already been explored in the context of signal classification. In addition to pattern recognition in ECG records [34,35], it has also been applied to RR records using a threshold to assign symbols to interbeat intervals [37]. Applied also to RR records, the method described in [38] represented heart rate accelerations by the symbol 1, and decelerations by 0. Even the number of forbidden words have been included as a classification feature [39]. However, these methods are too record specific, and their discriminating power was not very high [39].

**Figure 1:**
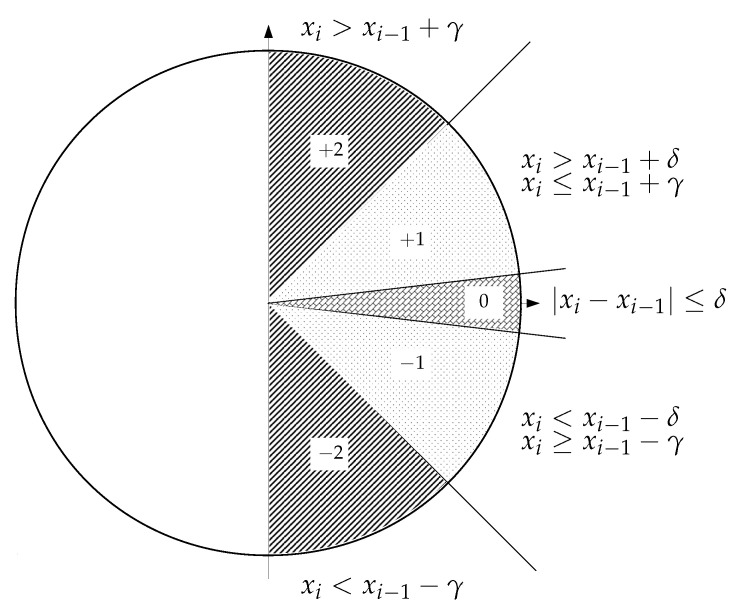
Graphical interpretation of the Slope Entropy (SlopEn) approach using three levels. Possible symbols are +2, +1, 0, −1, and −2, depending on the amplitude difference between consecutive samples.

We wanted to apply a similar scheme that had to be simple and less dependent on thresholds and specific amplitude values or records. In this regard, thresholds were based on the gradient between consecutive samples, instead of absolute values, and second, symbols should be assigned according to a certain range of differences or slopes, as depicted in Figure 1.

Thus, each subsequence of length *m* drawn from x, can be transformed into another subsequence of length m−1 with the differences of each pair of consecutive samples, $x_{i}-x_{i-1}$. Then, a threshold or thresholds must be applied to these differences in order to find the corresponding symbolic representation. Once these symbols are obtained, a Shannon entropy approach can be applied in a similar manner as for PE, but instead of normalising by a constant value (the possible number of ordinal patterns in PE, m!), the factor employed corresponds to the actual number of slope patterns found. This way, the possible additional information provided by forbidden patterns [40,41], can be also exploited, although the values obtained can not be considered a true probability value.

The specific SlopEn configuration proposed in the present study is very straightforward. It considers the horizontal increment between consecutive samples to always be 1, and the differences (vertical increment) are thresholded by a parameter γ, taken as 1 in the present study ($45^{\circ }$ angle). The vicinity of the 0–difference region is managed by another threshold, termed δ, whose chosen value in this case was $1\times 10^{-3}$, to account for possible ties [26]. Being $x_{i}$ and $x_{i-1}$ two consecutive values of the input time series, the symbols can be assigned according to the following rules:

- If $x_{i}>x_{i-1}+\gamma$, the symbol is +2.
- If $x_{i}>x_{i-1}+\delta$ and $x_{i}\leq x_{i-1}+\gamma$, below the $45^{\circ }$ angle and above the 0 region when γ=1, the symbol is +1.
- In the vicinity of the 0 difference, when $\left|x_{i}-x_{i-1}\right|\leq \delta$, the symbol assigned is 0.
- $x_{i}<x_{i-1}-\delta$ and $x_{i}\geq x_{i-1}-\gamma$, above the $-45^{\circ }$ angle, and below the 0 region when γ=1, the symbol is −1.
- If $x_{i}<x_{i-1}-\gamma$, the symbol is −2.

In any method, a resampling of the input sequence can be applied to study the possible information distribution across other temporal scales. The parameter that accounts for the magnitude of this new scale, the embedded delay, is usually represented by τ, with τ≥1. In this work, τ=1 for all the experiments, since it is still the most frequent case [29], except in Section 3.5.

For example, a subsequence $\left\{4.4,3.6,5.3\right\}$ would result in a vector of differences $\left\{-0.8,1.7\right\}$, whose SlopEn symbolic representation is $\psi_{j=0}^{3}=\left\{-1,+2\right\}$, with vector components $\psi_{0}=-1$ and $\psi_{1}=+2$. There is a complete and detailed SlopEn computation example in Appendix A, with source code in Appendix B. All the SlopEn computation steps are listed in Algorithm 1.

**Algorithm 1 **Slope Entropy (SlopEn) Algorithm **Input:** Time series x, embedded dimension m>2, length N>m+1, δ, γ>δ **Initialisation:** SlopEn ←0, slope pattern counter vector $c\leftarrow \left\{⌀\right\}$, slope patterns relative frequency vector $p\leftarrow \left\{⌀\right\}$, list of slope patterns found $\Psi^{m}\leftarrow \left\{⌀\right\}$

**for** j←0,…,N−m **do**
    **for** i←j+1,…,j+m−1 **do**        **if** $\left(x_{i}-x_{i-1}\right)\in \left[-\delta ,\delta \right]$ **then**
           $\psi_{i-(j+1)}\leftarrow$ 0        **end if**        **if** $\left(x_{i}-x_{i-1})\in ]\delta ,\gamma \right]$ **then**           $\psi_{i-(j+1)}\leftarrow$ +1        **end if**        **if** $\left(x_{i}-x_{i-1}\right)\in ]\gamma ,\infty [$ **then**           $\psi_{i-(j+1)}\leftarrow$ +2        **end if**        **if** $\left(x_{i}-x_{i-1}\right)\in [-\gamma ,-\delta [$ **then**           $\psi_{i-(j+1)}\leftarrow$ −1        **end if**        **if** $\left(x_{i}-x_{i-1}\right)\in ]-\infty ,\gamma [$ **then**           $\psi_{i-(j+1)}\leftarrow$ −2        **end if**    **end for**    bFound ← **false**
    **for** $i\leftarrow 0,\ldots ,\mathrm{sizeof}\left(\Psi^{m}\right)-1$ **do**        **if** $\psi_{j}^{m}=\Psi_{i}^{m}$ **then**           $c_{i}\leftarrow c_{i}+1$           bFound ← **true**           **break**
        **end if**    **end for**    **if not** bFound **then**        $\Psi^{m}⇐\psi_{j}^{m}$        c⇐1    **end if**
**end for**

**for** $i\leftarrow 0,\ldots ,\mathrm{sizeof}\left(\Psi^{m}\right)-1$ **do**
    $p⇐p_{i}\leftarrow \frac{c_{i}}{\mathrm{sizeof}\left(\Psi^{m}\right)}$    SlopEn ← SlopEn+$\left(-p_{i}\log p_{i}\right)$
**end for**


▹ For all the samples in the input time series ▹ Examine samples in a subsequence of length *m*
 ▹ Add symbol 0 to pattern vector $\psi_{j}^{m}$  ▹ Add symbol +1 to pattern vector $\psi_{j}^{m}$  ▹ Add symbol +2 to pattern vector $\psi_{j}^{m}$  ▹ Add symbol −1 to pattern vector $\psi_{j}^{m}$  ▹ Add symbol −2 to pattern vector $\psi_{j}^{m}$   ▹ Search on list of patterns already found for current pattern $\psi_{j}^{m}$▹ A match for $\psi_{j}^{m}$ found in $\Psi_{i}^{m}$▹ Update frequency counter for $\psi_{j}^{m}$ ▹ Stop search   ▹ Slope pattern $\psi_{j}^{m}$ not found in $\Psi_{i}^{m}$▹ Append pattern $\psi_{j}^{m}$ to list $\Psi_{i}^{m}$▹ Append and initialise pattern count    ▹ Append probability normalised by number of actual patterns found ▹ Compute Shannon entropy  
 **Output:** SlopEn$\left(x,m,N,\gamma ,\delta \right)$                                                   ▹ Return result  

Algorithm 1 can easily be optimised. For example, consecutive slope patterns overlap in the last samples, and it would not be necessary to compute the entire pattern for each subsequence. However, the algorithm is proposed in a generic and basic form, and optimisations are left for future studies.

### 2.4. Experimental Dataset

**Figure 2:**
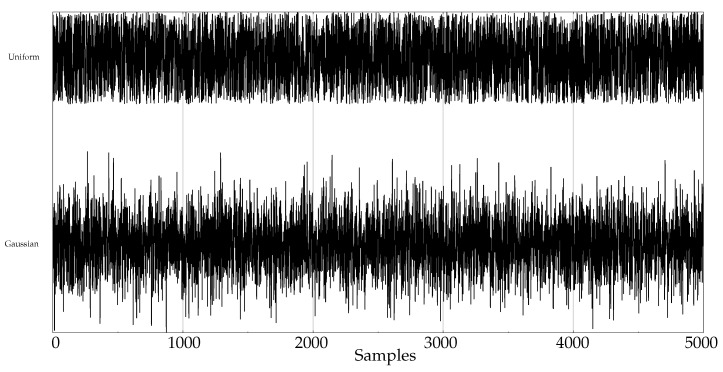
Example of generated synthetic records for the RANDOM database.

**Figure 3:**
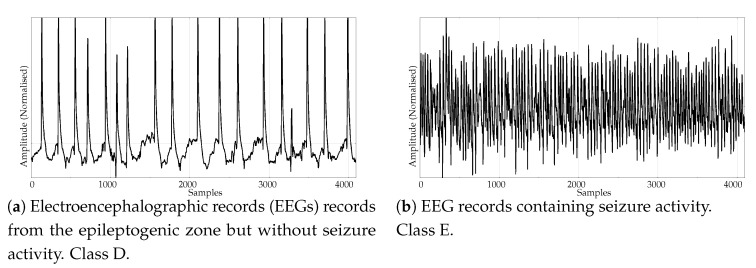
Examples of EEG records from the two classes used in the experiments.

**Figure 4:**
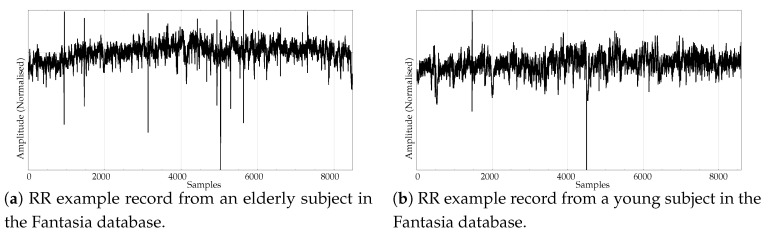
Examples of signals from the two classes of the RR database.

**Figure 5:**
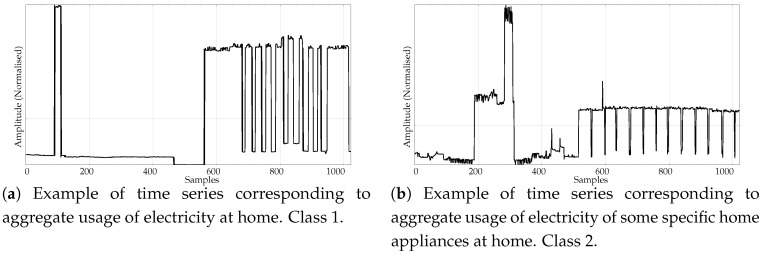
Example of time series from the two classes included in the ENERGY experimental dataset.

**Figure 6:**
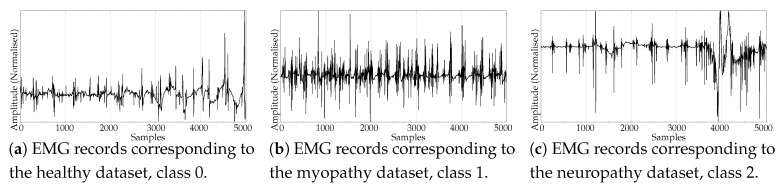
Examples of Electromyogram (EMG) records from the three classes used in the experiments.

The experimental dataset was composed of synthetic and real records. These datasets are described next:

- Random records. There is a clear synthetic case where PE failed to find differences between two classes: random time series with Gaussian or uniform amplitude distributions. This is a representative example of what happens when classes under analysis have the same temporal correlations but differ in amplitude: PE discriminating power gets lost [42]. A dataset of this case was included in the experiments in order to find out if SlopEn was capable of overcoming this known weakness of PE. Two classes were generated using Gaussian or uniform amplitude distributions, with 100 records each, with a length of 5000 samples. An example of records from each class is shown in Figure 2. This dataset will be referred to in the paper as the RANDOM dataset.
- Electroencephalographic records (EEGs) are the focus of many studies using entropy measures [43,44,45]. They have been used for a variety of purposes, such as to assess the mental status of a subject, driver’s fatigue, depth of anaesthesia, to detect a neurological disorder, or to predict the onset of epileptic seizures. There is also a great public availability of EEG records. For its good results using PE and SampEn in previous works, and due to the fact that it is probably the most widely known and analysed EEG database, we chose the University of Bonn EEG database [46]. There are five record classes in this database, but we only used the seizure–free and seizure–included records of classes D and E, respectively (100 records each one, uniform length of 4096 samples), easily separable, in principle. An example of class D record is plotted in Figure 3a, and in Figure 3b for class E.
- Another type of biomedical records extensively analysed using non–linear methods are series of time durations between consecutive R–waves in the electrocardiogram (ECG), or RR intervals [47,48,49]. We chose a publicly available RR database from the PhysioBank [50], the well known Fantasia database [51]. This database contains 20 young (21–34 years old) and 20 elderly (68–85 years old) healthy subjects data whose ECG signal was recorded during 120 min while in continuous supine resting. Examples of records from the elderly and young population are shown in Figure 4a,b, respectively.
- Entropy measures are also very popular in other time series domains, beyond the very successful one of biomedical records. Along this line, we looked for other publicly available datasets featuring a complete different kind of time series, and we found the varied and diverse repository at www.timeseriesclassification.com [52]. Within this repository, we chose two classes of data from the Personalised Retrofit Decision Support Tools for UK Homes Using Smart Home Technology (REFIT) project [53]. The first class contains data related to aggregate usage of electricity (Figure 5a), and the second one to aggregate usage of electricity of some specific home appliances (Figure 5b). This dataset contains 20 records from each class, with a uniform length of 1022 samples. We used this dataset in a previous study [33] where PE was unable to find significant differences between the two classes. Therefore, this should be considered a difficult dataset for entropy measures based only on ordinal patterns. We will refer to this dataset across the paper as the ENERGY dataset.
- The scientific and medical interest on Electromyograms (EMGs) and entropy measures is raising due to the recent availability of inexpensive continuous portable monitoring devices and the insight they provide into a number of important pathologies and motor disorders. They have been used to assess Parkinson’s disease [54], the neuromuscular impact of strokes [55], and muscular performance [56,57], to name just a few. The well–known site of Physionet [50] provides examples of EMGs, which we have used in previous classification studies, easily separable [22]. From three very long records of healthy, myopathy and neuropathy patients, we created three datasets by extracting non–overlapping epochs of 5000 samples. As a result, this dataset contains 10 healthy 5000 samples records (class 0), 22 myopathy 5000 samples records (class 1), and 29 neuropathy 5000 samples records (class 2). Examples of each class are shown in Figure 6a–c, respectively. This dataset will be referred to as the EMG dataset.

All records were normalised (zero mean and unit variance) before computing SampEn and PE. They were not normalised for SlopEn in order to assess the possible influence of the amplitude, given that the thresholds were constant and the same for all records. For example, differences in the RR dataset ranged mainly between 0 and 100 ms, between 100 and 200 μV for the EEG records, and between 0 and 20 μV for the EMG time series, and SlopEn should be able to deal with these differences. A normalised amplitude is less challenging in terms of input parameter dependence or configuration, and that will be analysed in future studies along with more specific guidelines for parameter selection.

## 3. Experiments and Results

### 3.1. Classification Accuracy Tests

This test was devised to find the classification accuracy achieved by each method using all datasets for *m* between 3 and 8. The average performance is shown in Table 1.

### 3.2. Embedded Dimension Influence Tests

**Figure 7:**
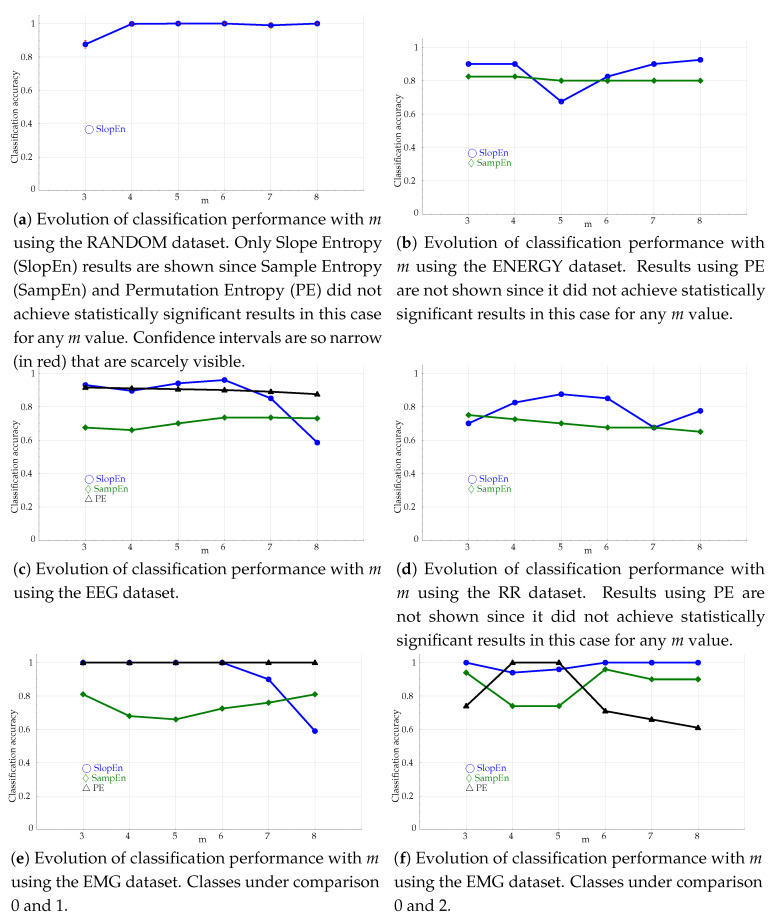
Evolution of classification performance with *m* for all the experimental datasets, and the three methods tested: PE, SampEn (r=0.25), and SlopEn.

The influence of *m* in the performance of PE and SampEn is a well known issue [58]. Although some efforts have been devoted to minimise this influence [59], it still plays an important role, and its impact should be characterised, and compared. To this end, the classification experiments were repeated for *m* values ranging from 3 up to 8. These results are plotted in Figure 7.

### 3.3. Length Influence Tests

**Figure 8:**
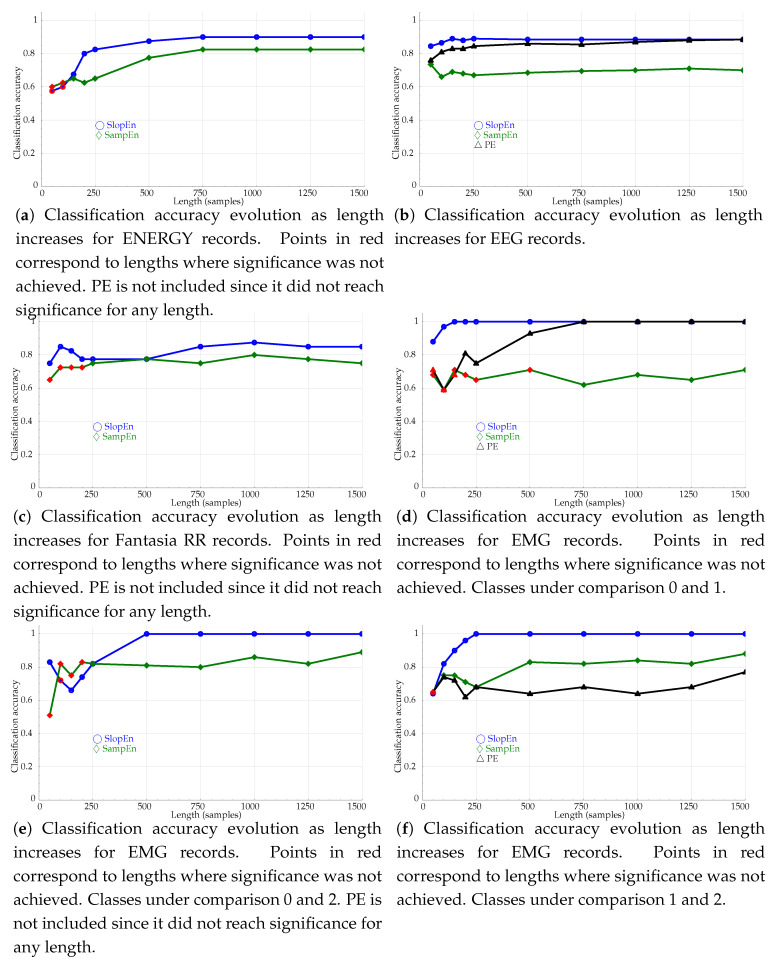
Classification accuracy evolution as length increases for the records in the dataset and the three entropy methods assessed, including the new method proposed, SlopEn (m=3). Red symbols represent statistically non–significant results. SampEn r=0.25.

The length influence was assessed using the classification accuracy achieved at lengths $n\in \left\{50,100,150,200,250,500,750,1000,1250,1500\right\}$. The first *n* samples of each record were used in these experiments instead of the entire records. The results are shown graphically in Figure 8.

### 3.4. Noise Influence Tests

**Figure 9:**
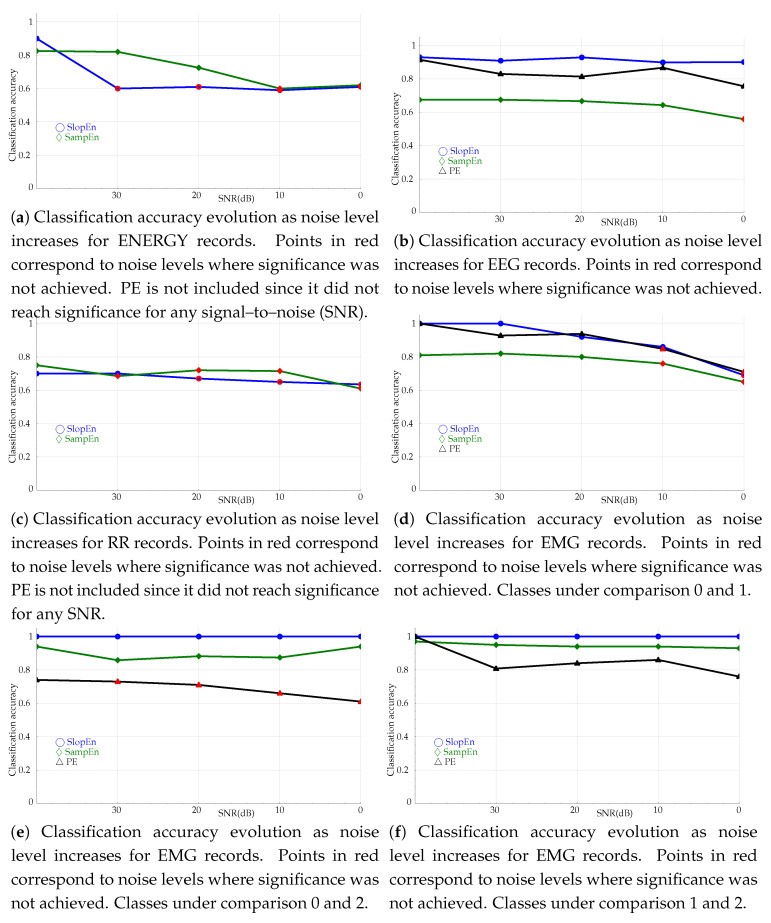
Classification accuracy evolution as noise level increases. The starting SNR, before the synthetic noise was added, was considered to be ∞. All the experiments used m=3. Confidence intervals are not shown due to their small size, around 0.002–0.003.

Robustness against noise has always been a very desirable property of non–linear measures. Some methods have failed to be widely used precisely because they were too sensitive to noise, no matter how high was their discriminating power when the records were clean. In order to avoid a similar fate for SlopEn, classification tests were repeated adding synthetic random uniform noise to the records in the experimental datasets, with signal–to–noise (SNR) ratios of 30dB, 20dB, 10dB, and 0dB, and m=3. It was not possible to know the initial SNR, therefore the synthetic SNR was computed against the baseline records, in its original state in the database. The evolution of the classification accuracy for each case is plotted in Figure 9.

### 3.5. Embedded Delay Influence Tests

The embedded delay τ in PE is frequently assumed to be 1 [29]. However, sometimes information about the time series dynamics is scattered across different time scales, and values τ>1, τ∈N, have been proven to be very useful in those cases [60]. That is also the case for SampEn [61].

**Figure 10:**
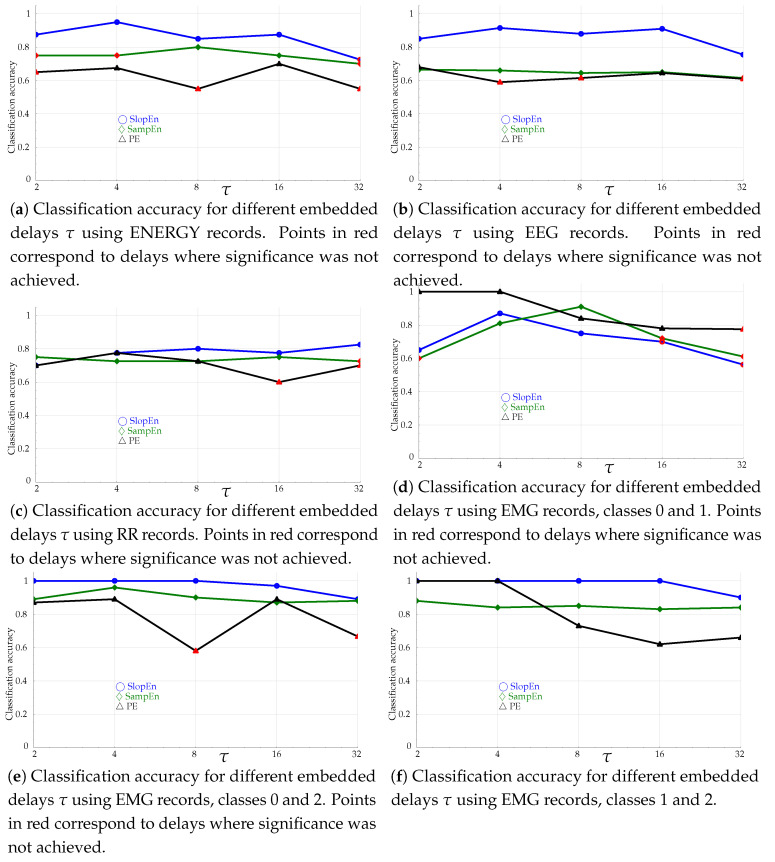
Classification accuracy as a function of embedded delay τ. All the experiments used m=3.

The experiments in this section were devised to assess the possible influence of τ on SlopEn, as it does on PE and SampEn. In practical terms, τ>1 corresponds to a downsampling process, where the output time series is obtained from the input one sampled at every τ samples, $x\left[nT\right]⟶x\left[n\tau T\right],n\;\in \;N^{+}$, but without applying a low–pass filter in order to avoid a possible aliasing [29]. The specific time scales employed in the experiments were $\left\{2,4,8,16,32\right\}$. For example, for τ=2, the initial data $\left\{x_{0},x_{1},x_{2},x_{3},x_{4},\ldots \right\}$ will become $\left\{x_{0},x_{2},x_{4},\ldots \right\}$. The results are shown in Figure 10.

### 3.6. SlopEn Parameters Influence Tests

The addition of more parameters to an entropy estimator method can be seen as a disadvantage, since, in principle, it increases the configuration effort for optimal performance. On the other hand, more parameters can also provide more flexibility for adaptation to the problem under analysis, provided the dependence on specific parameter values is low. This was the case, for example, for the evolution of SampEn to FuzzyEn [10]. The experiments in this section were devised to assess the robustness of SlopEn against changes in its specific parameters, γ and δ. The results are shown in Table 2.

## 4. Discussion

The classification accuracy achieved with SlopEn was the highest in all cases tested (Table 1). Since the datasets were chosen from previous works where PE exhibited some limitations due to its inability to include amplitude information [33,42], its results were the worst of the three metrics, as expected. PE only found significant differences for EEG and EMG records. On the contrary, SampEn and SlopEn classification accuracy was significant in all cases, with SampEn performing best for m=3, as usually recommended for SampEn and ApEn [4,36]. The performance for SlopEn was perfect for the RANDOM and EMG datasets, and very high for the other three. As hypothesised, SlopEn seems to take advantage of symbolic and amplitude information simultaneously, since it improves the individual results of both PE and SampEn. Cases where significant classification was not achieved in any experiment configuration were omitted in the subsequent tests.

The variation of this classification with the embedded dimension *m* yields a disparity of performances (Figure 7). In principle, SlopEn seems to have a greater variability with *m* than PE and SampEn, for datasets ENERGY (Figure 7b), EEG (Figure 7c), RR (Figure 7d), and the first case of the EMG dataset (Figure 7e). However, SlopEn is very stable for the RANDOM (Figure 7a) and the EMG dataset, second configuration (Figure 7f). Anyway, this is a similar behaviour exhibited by amplitude–based PE derived methods [33], and the performance of PE and SampEn is usually well below that of SlopEn for most of the *m* values tested, being SlopEn the only one achieving statistical significance in all cases.

It is important to note that the results in this paper can be compared with other in previous studies, since some experimental datasets are the same. For example, in [33], the classification performance of PE, along with improved PE versions, such as Weighted–PE [31], Fine Grained–PE [32], and Amplitude Aware–PE [29], was assessed too for datasets ENERGY, RR, and EEG. The best classification accuracy for these datasets in [33] was 0.87, achieved using Fine Grained–PE, 0.87, achieved using Weighted–PE, and 0.91, using Fine–Grained PE. The other method analysed in that study, Amplitude Aware–PE, achieved an accuracy of 0.62, 0.75, and 0.85 for those datasets. Using SlopEn, the results obtained in the present paper were 0.92, 0.87, and 0.96, using the same measure in the three cases. Therefore, SlopEn was able to outperform Weighted–PE, Fine Grained–PE, and Amplitude Aware–PE methods, too without customising the γ and δ parameters, or the number of thresholds.

The comparative results of the length analysis shown in Figure 8a–d indicate that SlopEn is reasonably robust against short datasets. The classification performance provided by PE has already been demonstrated to be robust in these terms [22], and SampEn has also been claimed to exhibit less dependence on length than other very successful methods like ApEn [58]. In this context of already robust methods, SlopEn was capable of outperforming them, with an even more stable behaviour, in addition to higher accuracy performances, discussed in other experiments. For Energy consumption records (Figure 8a), SlopEn stabilised at N=500, approximately, with performances above 0.8 at N=250. SampEn also needed some 500 samples, and PE was not able to achieve statistical significance for any length studied. The results in Figure 8b for EEG records show that the three methods become stable at N=250, but SlopEn achieves the maximum accuracy exhibited at N=1500, at that point already, whereas the other two still need more points. The stability for the classification of Fantasia RR records (Figure 8c) is quantitatively very similar among the three methods but not qualitatively since the results for the shortest lengths were not significant for SampEn, requiring at least 250 samples, and PE was unable to find significant differences. The experiment using EMG records is probably where the differences were most prominent (Figure 8d). With only 150 samples, SlopEn achieved the maximum performance, whereas PE needed 750 samples, the same for SampEn to achieve statistical significance. The last two length analysis experiments showed an even superior performance of SlopEn (Figure 8e,f).

SlopEn is also reasonably robust against noise. Except for the energy records (Figure 9a), the results achieved by SlopEn kept significance at lower SNR than SampEn and PE. For the EEG records (Figure 9b), the SlopEn response was quite flat, like that of PE, but with higher classification performance. SampEn lost significance at 0dB. In the case of RR results in Figure 9c, SampEn and SlopEn trends were fairly similar but significance was, again, better kept by SlopEn. It is also important to note that the experiments used m=3, at which SampEn achieved the maximum accuracy but not SlopEn (Figure 7d). With other *m* values, the SlopEn response was even better, but in order to maintain the homogeneity of the experiments, *m* was kept constant. EMG records exhibited the same behaviour, as can be seen in Figure 9d.

The embedded delay τ analysis results point in the same direction of SlopEn being superior to SampEn and PE. In all the cases tested and reported in Figure 10, the discriminating power of SlopEn was above that of the other two methods, except in the case of EMG records, classes 0 and 1, plotted in Figure 10d. Unless the time series exhibits a specific behaviour at a certain temporal scale, which is mainly not the case in the experimental dataset used in this study, the discriminating power is expected to fall with greater τ values since some signal information is lost when samples are removed. The response of SlopEn, along with that of SampEn, was reasonably flat in this regard, being PE response more oscillating. Moreover, there were several non–significant results for PE in all cases except Figure 10e,f, for SampEn except in Figure 10f. SlopEn only failed to find significant differences for τ=16,32 in Figure 10a,d. Although, as stated above, none of the records exhibited a clear temporal multiscale behaviour, it is important to note that methods that were unable to find differences for the baseline case, τ=1, were capable at other embedded delay values, such as PE with ENERGY (Figure 10a, τ=4,16) and RR records (Figure 10c, τ=2,4,8).

Table 2 shows the classification accuracy achieved for m=3 but with changes in SlopEn parameters γ and δ. Despite significant variations in these parameters, the performance was fairly stable, except for ENERGY records with great γ values. It seems that highest accuracy can be achieved provided δ is close to 0, and γ is relatively low, in the vicinity of 1 or 2. The key issue for SlopEn is arguably to distinguish between steep and gentle slopes in a fuzzy way, the exact values do not matter much. For example, the classification accuracy for RR records was the same, 0.70, for γ=1,3,5. For EEG records was almost constant (around 0.94) for all the different γ and δ combinations tested, and results for EMG dataset were also fairly stable, except for δ=1. ENERGY was one of the most difficult to classify cases, and that is reflected by a higher sensitivity to parameter values. This specific dataset required γ values below 4. It is also important to note that SlopEn was applied to not normalised records, with a great disparity in amplitudes. For optimal performance, a grid search could be conducted [14], and after a normalisation process, it could also be found out which parameter values are optimal for each dataset. Thus, further studies will be required to fine tune the use of the parameters, the number of thresholds, and to define a more uniform scheme using signal normalisation.

In a few tests where classification accuracies were not significant, this significance seemed sometimes not to be clearly correlated, or follow a uniform relationship pattern with classification accuracy. For example, in Figure 8e, using SlopEn, results were significant for length 50, with an accuracy of 0.83, then became non-significant for length 100, accuracy 0.72, and then significant again for length 150 and onward, with an accuracy at that point of 0.66, below the previous non-significant one of 0.72. Although this might seem counter-intuitive, it is actually relatively frequent due to the following reasons:

1. Accuracy is a kind of average between sensitivity and specificity, and a higher accuracy does not ensure significance because it can be the result of an unbalanced average. In this case, with a length of 100 samples, sensitivity was 0.51, and specificity 0.80, the average 0.72 was not significant because despite its high value, it came from a very low sensitivity. The same average for another test was achieved with a sensitivity of 0.80, and specificity of 0.68, but in this case, it was statistically significant. For length 150, the sensitivity was 1 and the specificity 0.57, significant for an accuracy of 0.66 but borderline.
2. There are many methods for equal mean hypothesis testing, each one with its strengths and weaknesses [62]. We used the Bootstrap method, since no assumptions about the input data have to be made [63]. However, the size and distribution of the data may influence its results, mainly when significance is borderline. For example, in the previous 0.72 and 0.66 example, the test prioritised specificity over sensitivity due to the size differences of the input classes, 10 and 29, respectively.
3. Rejecting the equal hypothesis is not a demonstration that it is completely false, or the other way round. Again, this is specially true in borderline cases where a minor random change can completely reverse the results.
4. There are many factors than can influence the differences between time series. They are usually considered stationary, but in reality, they might exhibit some temporal changes. For example, border effects are quite common in biomedical records [14], and this impacts the results in a length influence analysis. Other well–known effects are the stochastic resonance [64,65], whereby more noise does not necessary imply less discriminating power, just the opposite. Regarding the temporal scale given by τ, a regular trend should not be expected in all cases because the classification performance depends on the information content of the temporal scale analysed. These scales could be completely independent in terms of this information content.

## 5. Conclusions

We proposed in this paper a new entropy estimator termed Slope Entropy (SlopEn). It is based on the relative frequency of symbolic patterns, where each symbol is assigned according to the difference between consecutive samples of the input time series. The algorithm is very simple and easy to implement, with a lot of room for improvements and customisations in further studies.

Although SlopEn requires two new parameters, γ and δ, the classification accuracy is very stable for a wide range of these parameters but best for δ⟶0 and γ≈1,2. For normalised records, γ just needs to be rescaled in the range ]δ,1[, approximately. The goal is to somehow detect and account for abrupt differences between consecutive samples in the time series, combining the positional or ordinal information of these differences, and their magnitude: high (±2 symbol), low (±1 symbol), and ties (0 symbol).

A thorough and fair comparative study was conducted to assess the goodness of the new method proposed. Two of the most used entropy quantification methods were included in the experiments for comparative purposes: PE, as a good representative of ordinal–based approaches, and SampEn, based on amplitude differences. The experimental dataset included usual biomedical records in classification studies: EEG and RR records, records where PE achieved very good classification accuracy, EMG records, and synthetic and real records where PE has failed because amplitude information was a key distinguishing feature: Gaussian and uniform random noise [42], and energy consumption records [33]. This way, the study was also not constrained to just biomedical records.

In absolute terms, and using a default and stable parameter configuration for SlopEn, the classification accuracy achieved by this new measure was higher than that achieved by PE or SampEn. Even for the difficult cases where PE was unable to achieve statistically significant differences, SlopEn performance was between 87% and 100%. Not reported in this paper, but probably the focus of future studies, preliminary tests on other biomedical records, including RR, temperature, blood pressure, or blood glucose records, have shown the same trend of superior performance by SlopEn without any fine tuning at all, just using the baseline configuration proposed in the present study.

The additional tests for parameter dependence and noise robustness have also demonstrated that SlopEn is a very promising method for a myriad of classification applications in the near future and in different contexts. Specifically, SlopEn seems to be more dependent on *m* than PE or SampEn but yields a higher accuracy in most of the cases. On the contrary, SlopEn is more robust against length *N* than the other two methods, with significant results with just 50 samples in almost all cases tested. The dependence on its specific parameters, γ and δ, is low, and with a better customisation of these parameters, the performance of SlopEn in all tests would have been even higher. Anyway, normalisation should be considered a more robust approach, and in future works parameter customisation should be studied in the context of normalised records to ensure even better results. The embedded delay τ had a negligible influence on SlopEn for the temporal scales and records employed. SlopEn was also robust against different levels of noise.

Further studies will be required in order to find out the possible relationship between normalised amplitudes and thresholds for optimal performance. Moreover, the number of thresholds could also be characterised for a specific time series. Other works should be devised to design classifiers based on SlopEn in order to confirm its superior accuracy for a particular application and data. Due to its simplicity and good performance, SlopEn could become a successful and widespread entropy quantifier method similar to SampEn or PE.

## Figures and Tables

**Table 1:** Classification accuracy average results for all datasets. Statistically significant results are shown in bold. SampEn r=0.25.

Dataset	Accuracy		
	PE	SampEn	SlopEn
RANDOM	0.62±0.055	0.62±0.063	0.97±0.047
ENERGY	0.64±0.013	0.81±0.013	0.85±0.094
EEG	0.90±0.014	0.70±0.034	0.86±0.140
RR	0.62±0.031	0.69±0.036	0.78±0.081
EMG(0,1)	1±0.000	0.74±0.063	0.91±0.164
EMG(0,2)	0.78±0.171	0.86±0.098	0.98±0.026
EMG(1,2)	1±0.000	0.89±0.048	0.94±0.112

**Table 2:** SlopEn performance variation when parameters γ and δ are modified (m=3). The first column of data corresponds to the baseline configuration, that used in all the previous experiments.

γ	1	3	6	5	5	1	2
δ	$1\times 10^{-3}$	$1\times 10^{-3}$	$1\times 10^{-3}$	1	$1\times 10^{-3}$	$1\times 10^{-9}$	$1\times 10^{-9}$
RR	0.70	0.70	0.82	0.85	0.70	0.70	0.70
EEG	0.93	0.94	0.94	0.95	0.95	0.93	0.94
ENERGY	0.90	0.87	0.57	0.60	0.62	0.90	0.92
EMG	1,1,1	1,1,1	1,0.88,0.88	0.51,1,1	0.93,1,1	1,1,1	1,1,1

## Appendix A. Example of SlopEn Computation

Let x be an input time series series defined by $x=\left\{8.2\right.$, 8.1, 4.4, 3.6, 5.3, 5.4, 8.3, 1.9, 3.7, 8.6, 9.6, 9, 6, 8.7, 6.7, 3.3, 2, 2.5, 2.7, 4.6, 9.1, 1, 3.1, 1.7, 4.1, 3.8, 6.4, 1.3, 5.7, 3.4, 2.4, 2.1, $\left.4.2\right\}$. For m=3, N=33, and the default configuration described in Section 2.3, the computation of SlopEn is as follows:

- Extract first subsequence from x, $x_{j=0}^{3}=\left\{8.2,8.1,4.4\right\}$. Compute the corresponding slope pattern, $\psi_{j=0}^{3}=\begin{array}{c} \left\{-1,-2\right\} \end{array}$, since $8.1-8.2\in \left[-1,-0.001\right]$, and 4.4−8.1<−1. Append pattern $\left\{-1,-2\right\}$ to the list of patterns found $\Psi^{3}$ and initialise its counter to 1: $\Psi^{3}=\left\{\left\{\left\{-1,-2\right\},1\right\}\right\}$.
- The next subsequence from x is $x_{j=1}^{3}=\left\{8.1,4.4,3.6\right\}$. Pattern $\psi_{j=1}^{3}=\begin{array}{c} \left\{-2,-1\right\} \end{array}$. This pattern is not in $\Psi^{3}$. Append it, and initialise its counter to 1: $\Psi^{3}=\left\{\left\{\left\{-1,-2\right\},1\right\},\left\{\left\{-2,-1\right\},1\right\}\right\}$.
- Subsequence $x_{j=2}^{3}=\left\{4.4,3.6,5.3\right\}$. Pattern $\psi_{j=2}^{3}=\begin{array}{c} \left\{-1,2\right\} \end{array}$. This pattern is not in $\Psi^{3}$. Append it, and initialise its counter to 1: $\Psi^{3}=\left\{\left\{\left\{-1,-2\right\},1\right\},\left\{\left\{-2,-1\right\},1\right\},\left\{\left\{-1,2\right\},1\right\}\right\}$.
- Subsequence $x_{j=3}^{3}=\left\{3.6,5.3,5.4\right\}$. Pattern $\psi_{j=3}^{3}=\begin{array}{c} \left\{2,1\right\} \end{array}$. This pattern is not in $\Psi^{3}$. Append it, and initialise its counter to 1: $\Psi^{3}=\left\{\left\{\left\{-1,-2\right\},1\right\},\cdots ,\left\{\left\{-1,2\right\},1\right\},\left\{\left\{2,1\right\},1\right\}\right\}$.
- Subsequence $x_{j=4}^{3}=\left\{5.3,5.4,8.3\right\}$. Pattern $\psi_{j=4}^{3}=\begin{array}{c} \left\{1,2\right\} \end{array}$. This pattern is not in $\Psi^{3}$. Append it, and initialise its counter to 1: $\Psi^{3}=\left\{\left\{\left\{-1,-2\right\},1\right\},\cdots ,\left\{\left\{2,1\right\},1\right\},\left\{\left\{1,2\right\},1\right\}\right\}$.
- Subsequence $x_{j=5}^{3}=\left\{5.4,8.3,1.9\right\}$. Pattern $\psi_{j=5}^{3}=\begin{array}{c} \left\{2,-2\right\} \end{array}$. This pattern is not in $\Psi^{3}$. Append it, and initialise its counter to 1: $\Psi^{3}=\left\{\left\{\left\{-1,-2\right\},1\right\},\cdots ,\left\{\left\{1,2\right\},1\right\},\left\{\left\{2,-2\right\},1\right\}\right\}$.
- Subsequence $x_{j=6}^{3}=\left\{8.3,1.9,3.7\right\}$. Pattern $\psi_{j=6}^{3}=\begin{array}{c} \left\{-2,2\right\} \end{array}$. This pattern is not in $\Psi^{3}$. Append it, and initialise its counter to 1: $\Psi^{3}=\left\{\left\{\left\{-1,-2\right\},1\right\},\cdots ,\left\{\left\{2,-2\right\},1\right\},\left\{\left\{-2,2\right\},1\right\}\right\}$.
- Subsequence $x_{j=7}^{3}=\left\{1.9,3.7,8.6\right\}$. Pattern $\psi_{j=7}^{3}=\begin{array}{c} \left\{2,2\right\} \end{array}$. This pattern is not in $\Psi^{3}$. Append it, and initialise its counter to 1: $\Psi^{3}=\left\{\left\{\left\{-1,-2\right\},1\right\},\cdots ,\left\{\left\{-2,2\right\},1\right\},\left\{\left\{2,2\right\},1\right\}\right\}$.
- Subsequence $x_{j=8}^{3}=\left\{3.7,8.6,9.6\right\}$. Pattern $\psi_{j=8}^{3}=\begin{array}{c} \left\{2,1\right\} \end{array}$. This pattern is already in $\Psi^{3}$. Update its counter to 2: $\Psi^{3}=\left\{\left\{\left\{-1,-2\right\},1\right\},\cdots ,\left\{\left\{-1,2\right\},1\right\},\left\{\left\{2,1\right\},\begin{array}{c} 2 \end{array}\right\},\cdots \right\}$.
- Subsequence $x_{j=9}^{3}=\left\{8.6,9.6,9\right\}$. Pattern $\psi_{j=9}^{3}=\begin{array}{c} \left\{1,-1\right\} \end{array}$. This pattern is not in $\Psi^{3}$. Append it, and initialise its counter to 1: $\Psi^{3}=\left\{\left\{\left\{-1,-2\right\},1\right\},\cdots ,\left\{\left\{2,2\right\},1\right\},\left\{\left\{1,-1\right\},1\right\}\right\}$.
- Subsequence $x_{j=10}^{3}=\left\{9.6,9,6\right\}$. Pattern $\psi_{j=10}^{3}=\begin{array}{c} \left\{-1,-2\right\} \end{array}$. This pattern is already in $\Psi^{3}$. Update its counter to 2: $\Psi^{3}=\left\{\left\{\left\{-1,-2\right\},\begin{array}{c} 2 \end{array}\right\},\cdots \right\}$.
- Subsequence $x_{j=11}^{3}=\left\{9,6,8.7\right\}$. Pattern $\psi_{j=11}^{3}=\begin{array}{c} \left\{-2,2\right\} \end{array}$. This pattern is already in $\Psi^{3}$. Update its counter to 2: $\Psi^{3}=\left\{\left\{\left\{-1,-2\right\},2\right\},\cdots ,\left\{\left\{-2,2\right\},\begin{array}{c} 2 \end{array}\right\},\cdots \right\}$.
- Subsequence $x_{j=12}^{3}=\left\{6,8.7,6.7\right\}$. Pattern $\psi_{j=12}^{3}=\begin{array}{c} \left\{2,-2\right\} \end{array}$. This pattern is already in $\Psi^{3}$. Update its counter to 2: $\Psi^{3}=\left\{\left\{\left\{-1,-2\right\},2\right\},\cdots ,\left\{\left\{2,-2\right\},\begin{array}{c} 2 \end{array}\right\},\cdots \right\}$.
- Subsequence $x_{j=13}^{3}=\left\{8.7,6.7,3.3\right\}$. Pattern $\psi_{j=13}^{3}=\begin{array}{c} \left\{-2,-2\right\} \end{array}$. This pattern is not in $\Psi^{3}$. Append it, and initialise its counter to 1: $\Psi^{3}=\left\{\left\{\left\{-1,-2\right\},2\right\},\cdots ,\left\{\left\{1,-1\right\},1\right\},\left\{\left\{-2,-2\right\},1\right\}\right\}$.
- Subsequence $x_{j=14}^{3}=\left\{6.7,3.3,2\right\}$. Pattern $\psi_{j=14}^{3}=\begin{array}{c} \left\{-2,-2\right\} \end{array}$. This pattern is already in $\Psi^{3}$. Update its counter to 2: $\Psi^{3}=\left\{\left\{\left\{-1,-2\right\},2\right\},\cdots ,\left\{\left\{1,-1\right\},1\right\},\left\{\left\{-2,-2\right\},\begin{array}{c} 2 \end{array}\right\}\right\}$.
- Subsequence $x_{j=15}^{3}=\left\{3.3,2,2.5\right\}$. Pattern $\psi_{j=15}^{3}=\begin{array}{c} \left\{-2,1\right\} \end{array}$. This pattern is not in $\Psi^{3}$. Append it, and initialise its counter to 1: $\Psi^{3}=\left\{\left\{\left\{-1,-2\right\},2\right\},\cdots ,\left\{\left\{-2,-2\right\},2\right\},\left\{\left\{-2,1\right\},1\right\}\right\}$.
- Subsequence $x_{j=16}^{3}=\left\{2,2.5,2.7\right\}$. Pattern $\psi_{j=16}^{3}=\begin{array}{c} \left\{1,1\right\} \end{array}$. This pattern is not in $\Psi^{3}$. Append it, and initialise its counter to 1: $\Psi^{3}=\left\{\left\{\left\{-1,-2\right\},2\right\},\cdots ,\left\{\left\{-2,1\right\},1\right\},\left\{\left\{1,1\right\},1\right\}\right\}$.
- Subsequence $x_{j=17}^{3}=\left\{2.5,2.7,4.6\right\}$. Pattern $\psi_{j=17}^{3}=\begin{array}{c} \left\{1,2\right\} \end{array}$. This pattern is already in $\Psi^{3}$. Update its counter to 2: $\Psi^{3}=\left\{\left\{\left\{-1,-2\right\},2\right\},\cdots ,\left\{\left\{1,2\right\},\begin{array}{c} 2 \end{array}\right\},\cdots \right\}$.
- Subsequence $x_{j=18}^{3}=\left\{2.7,4.6,9.1\right\}$. Pattern $\psi_{j=18}^{3}=\begin{array}{c} \left\{2,2\right\} \end{array}$. This pattern is already in $\Psi^{3}$. Update its counter to 2: $\Psi^{3}=\left\{\left\{\left\{-1,-2\right\},2\right\},\cdots ,\left\{\left\{2,2\right\},\begin{array}{c} 2 \end{array}\right\},\cdots \right\}$.
- Subsequence $x_{j=19}^{3}=\left\{4.6,9.1,1\right\}$. Pattern $\psi_{j=19}^{3}=\begin{array}{c} 0,0,1\left\{2,-2\right\} \end{array}$. This pattern is already in $\Psi^{3}$. Update its counter to 3: $\Psi^{3}=\left\{\left\{\left\{-1,-2\right\},2\right\},\cdots ,\left\{\left\{2,-2\right\},\begin{array}{c} 3 \end{array}\right\},\cdots \right\}$.
- Subsequence $x_{j=20}^{3}=\left\{9.1,1,3.1\right\}$. Pattern $\psi_{j=20}^{3}=\begin{array}{c} \left\{-2,2\right\} \end{array}$. This pattern is already in $\Psi^{3}$. Update its counter to 3: $\Psi^{3}=\left\{\left\{\left\{-1,-2\right\},2\right\},\cdots ,\left\{\left\{-2,2\right\},\begin{array}{c} 3 \end{array}\right\},\cdots \right\}$.
- Subsequence $x_{j=21}^{3}=\left\{1,3.1,1.7\right\}$. Pattern $\psi_{j=21}^{3}=\begin{array}{c} \left\{2,-2\right\} \end{array}$. This pattern is already in $\Psi^{3}$. Update its counter to 4: $\Psi^{3}=\left\{\left\{\left\{-1,-2\right\},2\right\},\cdots ,\left\{\left\{2,-2\right\},\begin{array}{c} 4 \end{array}\right\},\cdots \right\}$.
- Subsequence $x_{j=22}^{3}=\left\{3.1,1.7,4.1\right\}$. Pattern $\psi_{j=22}^{3}=\begin{array}{c} \left\{-2,2\right\} \end{array}$. This pattern is already in $\Psi^{3}$. Update its counter to 4: $\Psi^{3}=\left\{\left\{\left\{-1,-2\right\},2\right\},\cdots ,\left\{\left\{-2,2\right\},\begin{array}{c} 4 \end{array}\right\},\cdots \right\}$.
- Subsequence $x_{j=23}^{3}=\left\{1.7,4.1,3.8\right\}$. Pattern $\psi_{j=23}^{3}=\begin{array}{c} \left\{2,-1\right\} \end{array}$. This pattern is not in $\Psi^{3}$. Append it, and initialise its counter to 1: $\Psi^{3}=\left\{\left\{\left\{-1,-2\right\},2\right\},\cdots ,\left\{\left\{1,1\right\},1\right\},\left\{\left\{2,-1\right\},1\right\}\right\}$.
- Subsequence $x_{j=24}^{3}=\left\{4.1,3.8,6.4\right\}$. Pattern $\psi_{j=24}^{3}=\begin{array}{c} \left\{-1,2\right\} \end{array}$. This pattern is already in $\Psi^{3}$. Update its counter to 2: $\Psi^{3}=\left\{\left\{\left\{-1,-2\right\},2\right\},\cdots ,\left\{\left\{-1,2\right\},\begin{array}{c} 2 \end{array}\right\},\cdots \right\}$.
- Subsequence $x_{j=25}^{3}=\left\{3.8,6.4,1.3\right\}$. Pattern $\psi_{j=25}^{3}=\begin{array}{c} \left\{2,-2\right\} \end{array}$. This pattern is already in $\Psi^{3}$. Update its counter to 5: $\Psi^{3}=\left\{\left\{\left\{-1,-2\right\},2\right\},\cdots ,\left\{\left\{2,-2\right\},\begin{array}{c} 5 \end{array}\right\},\cdots \right\}$.
- Subsequence $x_{j=26}^{3}=\left\{6.4,1.3,5.7\right\}$. Pattern $\psi_{j=26}^{3}=\begin{array}{c} \left\{-2,2\right\} \end{array}$. This pattern is already in $\Psi^{3}$. Update its counter to 5: $\Psi^{3}=\left\{\left\{\left\{-1,-2\right\},2\right\},\cdots ,\left\{\left\{-2,2\right\},\begin{array}{c} 5 \end{array}\right\},\cdots \right\}$.
- Subsequence $x_{j=27}^{3}=\left\{1.3,5.7,3.4\right\}$. Pattern $\psi_{j=27}^{3}=\begin{array}{c} \left\{2,-2\right\} \end{array}$. This pattern is already in $\Psi^{3}$. Update its counter to 6: $\Psi^{3}=\left\{\left\{\left\{-1,-2\right\},2\right\},\cdots ,\left\{\left\{2,-2\right\},\begin{array}{c} 6 \end{array}\right\},\cdots \right\}$.
- Subsequence $x_{j=28}^{3}=\left\{5.7,3.4,2.4\right\}$. Pattern $\psi_{j=28}^{3}=\begin{array}{c} \left\{-2,-1\right\} \end{array}$. This pattern is already in $\Psi^{3}$. Update its counter to 2: $\Psi^{3}=\left\{\left\{\left\{-1,-2\right\},1\right\},\left\{\left\{-2,-1\right\},\begin{array}{c} 2 \end{array}\right\}\cdots \right\}$.
- Subsequence $x_{j=29}^{3}=\left\{3.4,2.4,2.1\right\}$. Pattern $\psi_{j=29}^{3}=\begin{array}{c} \left\{-1,-1\right\} \end{array}$. This pattern is not in $\Psi^{3}$. Append it, and initialise its counter to 1: $\Psi^{3}=\left\{\left\{\left\{-1,-2\right\},2\right\},\cdots ,\left\{\left\{2,-1\right\},1\right\},\left\{\left\{-1,-1\right\},1\right\}\right\}$.
- Subsequence $x_{j=30}^{3}=\left\{2.4,2.1,4.2\right\}$. Pattern $\psi_{j=30}^{3}=\begin{array}{c} \left\{-1,2\right\} \end{array}$. This pattern is already in $\Psi^{3}$. Update its counter to 3: $\Psi^{3}=\left\{\left\{\left\{-1,-2\right\},2\right\},\cdots ,\left\{\left\{-1,2\right\},\begin{array}{c} 3 \end{array}\right\},\cdots \right\}$.

Once all the patterns have been processed, the resulting list of coincidences is $\left\{2,2,3,2,2,6,5,2,1,2,1,1,1,1\right\}$. Normalising by the number of actual patterns found, 14, and applying Shannon entropy, the final SlopEn value for this time series is 5.29.

## Appendix B. SlopEn Source Code Implementation

This section includes a possible implementation of the SlopEn algorithm using C++ programming language (no optimisation, no error checking). It is implemented using a base–2 logarithm, but it can be replaced by a natural logarithm. This source is included below:
